# Respiratory symptoms and exercise responses among adult E‐cigarette users with and without obesity

**DOI:** 10.14814/phy2.70993

**Published:** 2026-06-26

**Authors:** Joseph W. Walsh, Michael Beck, Alice Hinton, Theodore L. Wagener, Jason E. Lang, Jing G. Wang, Dharini M. Bhammar

**Affiliations:** ^1^ Center for Tobacco Research The Ohio State University Comprehensive Cancer Center, The Ohio State University Columbus Ohio USA; ^2^ Division of Medical Oncology, Department of Internal Medicine, College of Medicine The Ohio State University Wexner Medical Center Columbus Ohio USA; ^3^ Department of Pediatrics Duke University School of Medicine Durham North Carolina USA; ^4^ Duke Clinical Research Institute Duke University School of Medicine Durham North Carolina USA; ^5^ Division of Pulmonary Critical Care Medicine, Department of Internal Medicine, College of Medicine The Ohio State University Wexner Medical Center Columbus Ohio USA

**Keywords:** breathlessness, cardiopulmonary exercise test, dyspnea, electronic nicotine delivery system, lung function

## Abstract

Despite the high prevalence of both e‐cigarette use and obesity among young adults, little is known about whether obesity worsens lung function, ventilatory constraints, or respiratory symptoms among e‐cigarette users. The purpose of this study was to investigate the effects of obesity on lung function, ventilatory constraints, and exertional breathlessness in young adult e‐cigarette users. Forty‐seven 21–35‐year‐old e‐cigarette users (*n* = 19 with mild‐to‐moderate obesity) completed submaximal exercise testing (6 min at 40 W for females and 60 W for males followed by two 4‐min stages, each increased by 20 W increments; low, moderate, and higher work rates). Gas exchange, minute ventilation, operating lung volumes, and ratings of perceived breathlessness (RPB) and exertion (RPE) were assessed. Significant breathlessness was defined as RPB ≥3 (moderate or greater breathlessness). E‐cigarette users with obesity experienced a 13% higher oxygen uptake exercise, 15% greater minute ventilation, and 27% lower end‐expiratory lung volume, compared with those without obesity (*p* < 0.05). 74% of individuals with obesity and 64% of individuals without obesity reported moderate or greater breathlessness at the higher work rate. E‐cigarette use history and cardiopulmonary exercise testing could be considered during the evaluation of young adults with exertional breathlessness.

## 1 INTRODUCTION

E‐cigarette use increased from 4.5% in 2019 to 6.5% in 2023 among adults (Vahratian et al., 2025) and e‐cigarettes are now the most commonly used tobacco product among young adults, with 15.5% reporting regular use (Vahratian et al., 2025). E‐cigarette use has been associated with spirometric evidence of airway dysfunction, as well as increased respiratory symptoms (Chaffee et al., 2021; Darabseh et al., 2021; Joshi et al., 2021; Meo et al., 2018; Tackett et al., 2024; Tattersall et al., 2023; Wills et al., 2021). Young adults with obesity are more likely to initiate or regularly use e‐cigarettes, and obesity is highly prevalent in this age group, affecting 35.5% of adults between the ages of 20 and 39 (Delk et al., 2018; Lanza et al., 2017). Obesity exerts a mechanical load on the respiratory system, leading to reductions in functional residual capacity that predispose individuals to ventilatory constraints, such as expiratory flow limitation (EFL) and dynamic hyperinflation during exercise (Balmain et al., 2020; O'Donnell et al., 2012). When combined with the increased metabolic and ventilatory demands of obesity, these obesity‐related ventilatory constraints may exacerbate respiratory symptoms such as exertional breathlessness among e‐cigarette users with obesity (Balmain et al., 2020; O'Donnell et al., 2012). Exertional breathlessness can be a distressing symptom that can lead to exercise avoidance (Gilman & Banzett, 2009), contributing to reduced cardiorespiratory fitness, weight gain, and worsening cardiometabolic health (Bames et al., 2012). Despite the high prevalence of both e‐cigarette use and obesity among young adults, little is known about whether obesity worsens lung function, ventilatory constraints, or respiratory symptoms such as exertional breathlessness among e‐cigarette users.

Given that regular exercise is essential for weight management and exertional breathlessness may present a barrier to initiating and sustaining physical activity, it is critical to investigate exertional breathlessness and potential underlying mechanisms (i.e., changes in lung function and ventilatory constraints) among e‐cigarette users with obesity. Because resting pulmonary function testing cannot capture dynamic ventilatory mechanics and symptom development during exertion, submaximal exercise testing offers a useful approach for characterizing exertional breathlessness and cardiopulmonary responses during steady‐state exercise. This approach has not previously been applied to young adult e‐cigarette users with obesity. The objective of this study was to investigate the effects of obesity on lung function, ventilatory constraints, and exertional breathlessness during submaximal exercise in young adult e‐cigarette users. We hypothesized that obesity would be associated with reduced lung function and greater ventilatory constraints and exertional breathlessness.

## 2 METHODS

The data presented here were collected as part of a larger study that investigated the acute and long‐term respiratory effects of e‐cigarette use in young adults with and without obesity (NCT05869318). We include only the methods and data essential to the novel findings presented here.

### 2.1 Participants

Participants were recruited through flyers, emails, text messages, and social media advertisements targeting the general community, the Ohio State University student community, and from a pool of volunteers from prior e‐cigarette research studies who had consented to be recontacted for future research opportunities. Participants were 21–35 years old, free from any diagnosed cardiopulmonary or metabolic diseases, and were regular nicotine e‐cigarette users, defined as daily use for at least 3 months prior to enrollment. Participants were excluded if they used tobacco products other than e‐cigarettes or used cannabis products >10 days in the past month. Participants were excluded if they were competitive athletes (to avoid including highly fit individuals) or actively trying to quit e‐cigarette use. Two groups of participants were enrolled: those without obesity (body mass index (BMI) = 18.5–24.9 kg/m^2^) and those with mild‐to‐moderate obesity (BMI = 30–50 kg/m^2^); overweight participants (BMI = 25–29.9 kg/m^2^) were excluded to yield two discrete groups for analysis.

The protocol was reviewed and approved by The Ohio State University (OSU) Cancer Institutional Review Board (approval no. 2022C0202). The study was conducted in accordance with the Declaration of Helsinki. Study procedures were completed at the OSU Center for Tobacco Research, Columbus, OH from April 2023 to May 2024. All participants provided written, informed consent.

### 2.2 Study procedures

Participants were asked not to consume any caffeine for 6 h or a heavy meal for 2 h prior to the visit. Participants completed a product use history questionnaire to determine ever and current use, use patterns, age of initiation, and regular use of e‐cigarettes. Survey data were collected and managed using REDCap electronic data capture tools hosted at OSU (Harris et al., 2009; Harris et al., 2019). Height and weight were measured using a stadiometer and a calibrated digital weighing scale, respectively. On the same day, participants completed a pulmonary function test and a submaximal constant‐load exercise test, which are described below.

### 2.3 Pulmonary function testing

All participants had spirometry, lung volumes, diffusing capacity, and maximal voluntary ventilation determinations using a body plethysmograph (Platinum Elite, MGC Diagnostics, Saint Paul, MN, USA) according to American Thoracic Society guidelines (Macintyre et al., 2005; Miller et al., 2005; Wanger et al., 2005). Published reference equations were used to calculate predicted values (Burrows et al., 1961; Cooper et al., 2017; Goldman & Becklake, 1959).

### 2.4 Exercise testing

A submaximal exercise test was completed on a cycle ergometer (Lode Corival) approximately 15 min after completing the pulmonary function test. The goal of the test was to evaluate cardiorespiratory responses and exertional breathlessness across a range of work rates that would be tolerable for participants (Babb et al., 2008; Bernhardt et al., 2022). After 4 min of rest, participants cycled at three consecutive constant work rates: 40 W (6 min), 60 W (4 min), and 80 W (4 min) for females, and 60 W (6 min), 80 W (4 min), and 100 W (4 min) for males. The selected work rates were informed by the average ventilatory threshold work rates reported in adults with and without obesity (110 W for males and 82 W for females) (Wong et al., 2021) and were comparable to the submaximal work rates used in a prior study of adults with obesity (90 W for males and 60 W for females) (Bernhardt et al., 2019; Bernhardt & Babb, 2014). Minute ventilation (V̇_E_), gas exchange (V̇O_2_ and V̇CO_2_), and breathing pattern were measured continuously (Ultima CPX, MGC Diagnostics, St. Paul, MN, USA). Heart rate was measured continuously (Nellcor PM100N, Medtronic, Minneapolis, MN, USA).

### 2.5 Exertional symptoms

Borg ratings of perceived breathlessness (RPB: 0–10 scale) and exertion (RPE: 6–20 scale) were assessed every 2 min during exercise (Borg, 1970, 1982); ratings obtained in the last minute of each work rate were used for analysis (Bernhardt et al., 2022; Bhammar et al., 2016). A standardized script was used to anchor ratings prior to each test. A multidimensional dyspnea profile was completed after exercise using 1–10 scales to assess unpleasantness and negative affective responses (i.e., depression, anxiety, frustration, anger, fear) related to breathlessness and to rate 15 breathlessness descriptors (Banzett et al., 2015; Mahler et al., 1996).

### 2.6 Operating lung volumes and ventilatory constraints

Two inspiratory capacity (IC) maneuvers were performed 1 min apart at rest and during the last 2 min of each exercise work rate, with participants inhaling maximally to total lung capacity (TLC). If the two IC measures were within 0.15 L, they were averaged; otherwise the higher value was recorded. End‐expiratory lung volume (EELV) was calculated as TLC − IC and end‐inspiratory lung volume (EILV) was calculated as EELV + V_T_. EFL was calculated as the percentage of tidal volume (%V_T_) where tidal expiratory flow exceeded maximal expiratory flow (Milic‐Emili, 2000). Any overlap >0% was recorded as EFL (Strozza et al., 2020). Dynamic hyperinflation was calculated as the change in IC from rest to each exercise work rate. A participant was classified as experiencing dynamic hyperinflation if the decrease in IC was ≥150 mL (O'Donnell et al., 2001).

### 2.7 Statistical analysis

A sample size of 48 participants would provide 80% power for a two‐sample *t*‐test to detect differences in continuous outcome measures between participants with and without obesity assuming an effect size of Cohen's *d* ≥ 0.77 and a significance level of 0.05. Results are expressed as mean ± standard deviation (SD) unless otherwise specified and, as exercise work rates differed between males and females, results were presented by both obesity group and sex. Normality of the data was assessed using the Shapiro–Wilk test, and rank transformations were completed prior to analysis for variables that were not normally distributed (Conover & Iman, 1981). General linear models were used to examine group (with vs. without obesity) and sex (male vs. female). Linear mixed models were used to examine differences during exercise based on group (with vs. without obesity), sex (male vs. female), exercise work rate (low, moderate, and high), and all interactions. Residuals were modeled with an AR(1) structure to account for correlations between observations from the same participant. Tukey's adjustment was applied to post hoc comparisons. Chi‐square or Fisher's exact tests were used to detect proportional differences in categorical variables. Odds ratios were calculated using GraphPad Prism (v10.6.0, Dotmatics, Boston, MA, USA) while SAS (v9.4, Cary, NC, USA) was used for all other analyses. *p* < 0.05 was considered statistically significant and all tests were two‐sided.

## 3 RESULTS

Fifty‐six volunteers provided written informed consent, 9 of whom did not meet eligibility criteria. Data from 47 participants were included in the analysis. Participants reported 17 different brands of e‐cigarettes, with Breeze, Elfbar, Vuse, Lost Mary, and Geek being the most common. Fruity flavors were the most common (66%), followed by menthol (21%) and fruit‐ice/fruit‐mint (11%). Only 1 participant was using tobacco‐flavored vapes. Ninety percent of participants were using 5% nicotine strength e‐cigarettes, with the remaining using 2.4%–3%. Participants reported an average of 134 e‐cigarette puffs taken per day (95% confidence interval [CI]: 10–500). Males with obesity were older than those without obesity and reported initiating e‐cigarette use approximately 5 years later than males without obesity (Table 1). Individuals with obesity reported a greater number of daily e‐cigarette puffs compared with those without obesity (Table 1).

**TABLE 1:** Participant characteristics, e‐cigarette use history, and pulmonary function measures reported as mean ± SD unless otherwise specified.

	Without obesity		With obesity		*p*‐value		
	Female	Male	Female	Male	Interaction	Main effect	
					Group × Sex	Group	Sex
*N*	10	18	5	14			
Age	25.4 ± 3.6	23.3 ± 3.1	25.4 ± 3.1	29.0 ± 4.5	0.0223	0.0209	0.5307
Height (cm)	163.3 ± 7.8	177.4 ± 6.5	166.5 ± 3.5	176.6 ± 4.7	0.3154	0.5530	<0.0001
Weight (kg)	57.4 ± 7.4	69.3 ± 8.4	103.0 ± 17.8	109.5 ± 15.3	0.4944	<0.0001	0.0218
BMI (kg/m^2^)	21.97 ± 2.4	21.97 ± 1.87	37.08 ± 5.94	35.04 ± 3.94	0.3412	<0.0001	0.3408
*E‐cigarette use*							
Age at first use (years)	19.3 ± 4.4	17.2 ± 2.9	20.2 ± 4.0	22.1 ± 4.1	0.1103	0.0222	0.9325
Age began regular use (years)	21.2 ± 5.1	18.4 ± 2.9	21.2 ± 4.0	24.9 ± 5.0	0.0242	0.0242	0.7441
# Puffs per day; Median (IQR)	73 (140)	76 (55)	150 (200)	100 (160)	0.1744	0.0303	0.2428
# Days used in past 30 days	27 ± 5	29 ± 2	29 ± 2	27 ± 6	0.1675	0.8993	0.7592
Duration of use (years)	4.18 ± 2.86	4.84 ± 2.25	4.21 ± 3.03	4.17 ± 2.38	0.6700	0.6984	0.7068
*Pulmonary function*							
FVC (L)	4.11 ± 0.51	5.40 ± 0.69	4.16 ± 0.49	5.63 ± 0.57	0.6556	0.4888	<0.0001
FVC (%pred)	110 ± 9	101 ± 10	103 ± 8	107 ± 10	0.0516	0.8818	0.4942
FEV_1_ (L)	3.29 ± 0.53	4.32 ± 0.56	3.54 ± 0.41	4.39 ± 0.56	0.5992	0.3782	<0.0001
FEV_1_ (%pred)	102 ± 11	96 ± 10	102 ± 9	100 ± 10	0.5377	0.4472	0.2408
FEV_1_/FVC (%)	79.9 ± 8.2	80.1 ± 6.0	85.0 ± 2.6	77.9 ± 6.3	0.0858	0.4966	0.1103
PEF (%pred)	91 ± 14	84 ± 12	98 ± 17	92 ± 20	0.9838	0.1475	0.2106
FEF_25‐75_ (%pred)	85 ± 22	84 ± 18	98 ± 23	90 ± 21	0.5642	0.1558	0.4845
MVV (L/min)	109 ± 14	147 ± 29	130 ± 27	156 ± 36	0.5365	0.1222	0.0013
MVV (%pred)	97 ± 8	79 ± 15	113 ± 23	88 ± 19	0.4980	0.0248	0.0002
TLC (L)	5.35 ± 0.72	6.79 ± 0.83	5.07 ± 0.57	7.08 ± 0.64	0.2453	0.9876	<0.0001
TLC (%pred)	103 ± 5	95 ± 11	93 ± 9	102 ± 10	0.0113	0.6439	0.8225
IC (L)	2.34 ± 0.41	3.11 ± 0.46	2.93 ± 0.35	4.15 ± 0.57	0.1552	<0.0001	<0.0001
IC (%pred)	95 ± 20	85 ± 10	111 ± 10	118 ± 16	0.0836	<0.0001	0.7658
FRC (%TLC)	57.0 ± 7.6	55.6 ± 5.3	45.3 ± 4.9	44.0 ± 4.8	0.9768	<0.0001	0.4700
DL_CO_/V_A_ (%pred)	108 ± 14	105 ± 11	115 ± 13	105 ± 10	0.3409	0.3008	0.0812

Abbreviations: BMI, body mass index; DL_CO_/V_A_, diffusion capacity of the lung for carbon monoxide/alveolar volume; FEF_25‐75_: forced expiratory flow between 25% and 75% of FVC; FEV_1_, forced expiratory volume in 1s; FRC, functional residual capacity; FVC, forced vital capacity; IC, inspiratory capacity; MVV, maximal voluntary ventilation; PEF, peak expiratory flow; TLC, total lung capacity.

### 3.1 Pulmonary function

All participants demonstrated normal forced vital capacity (FVC) and lung diffusion capacity with values that were ≥80% predicted. Only 1 participant (a male without obesity) had a forced expiratory volume in 1 s (FEV_1_) below 80% of predicted. Thirty‐four percent of participants had mid‐expiratory flows (forced expiratory flow between 25% and 75% of FVC; FEF_25‐75_) below 80% of predicted, including 39% of individuals without obesity and 26% of individuals with obesity, with no group differences (*p* = 0.3571; chi squared test).

There were no significant differences in spirometric or diffusion capacity measures between individuals with and without obesity (Table 1). On average, males had larger absolute lung volumes (FVC, TLC) and maximum voluntary ventilation (MVV) (Table 1). Individuals with obesity had higher MVV (%predicted) and IC, and lower functional residual capacity (FRC, % of TLC) than their normal‐weight counterparts (Table 1).

### 3.2 Exercise responses

Because exercise work rates were different for males and females, results are presented separately for males and females, with obesity status shown for each sex in Tables 2 and 3. Despite sex‐specific exercise work rates, V̇_E_ expressed as a percentage of MVV did not differ by sex, indicating similar relative ventilatory demand during the three exercise levels for males and females (Table 2). On average, individuals with obesity experienced a 13% higher metabolic cost of exercise (i.e., V̇O_2_; *p* < 0.001) and 15% greater ventilatory demand (i.e., V̇_E_; *p* = 0.0057) compared with those without obesity (Table 2). V_T_ was 23% higher in individuals with obesity (*p* = 0.0044) compared with those without obesity, while breathing frequency was not different (*p* = 0.8990) (Table 2). Despite exercising at a lower absolute work rate at the higher exercise level (80 W vs. 100 W), females exhibited a higher heart rate response compared with males (*p* = 0.0113 for heart rate and *p* = 0.0271 for heart rate as a percentage of predicted maximum heart rate; Table 2). Females, on average, exhibited a higher V̇_E_/V̇CO_2_ (*p* = 0.0013) and lower partial pressure of end‐tidal carbon dioxide (P_ET_CO_2_) during exercise (*p* = 0.0080) compared with males (Table 2).

**TABLE 2:** Cardiopulmonary exercise responses at rest and during low, moderate (mod), and higher work rates for males and females with and without obesity reported as mean ± standard error (SEM).

	Sex	Group	WR (Watt)	HR (beats/ min)	HR (%Pred HR_max_)	V̇O_2_ (L/min)	RER	V̇_E_ (L/min)	V̇_E_ (%MVV)	*f*_B_ (breaths/min)	V_T_ (L)	V̇_E_/V̇CO_2_	P_ET_CO_2_ (mmHg)	RPB	RPE
Rest	F	Without Obesity		84 ± 5	43 ± 2	0.27 ± 0.03	0.82 ± 0.03	9.6 ± 1.7	9 ± 2	15 ± 1	0.66 ± 0.11	43.3 ± 1.1	30 ± 1	0.1 ± 0.5	‐
		With Obesity		95 ± 7	49 ± 4	0.34 ± 0.04	0.86 ± 0.05	11.9 ± 2.4	10 ± 3	16 ± 2	0.74 ± 0.16	40.8 ± 1.5	32 ± 2	0.1 ± 0.6	‐
	M	Without Obesity		77 ± 3	39 ± 2	0.33 ± 0.02	0.91 ± 0.02	11.3 ± 1.3	8 ± 1	13 ± 1	0.94 ± 0.08	37.2 ± 0.8	35 ± 1	0.6 ± 0.3	‐
		With Obesity		80 ± 4	42 ± 2	0.41 ± 0.02	0.97 ± 0.03	15.9 ± 1.5	10 ± 2	14 ± 1	1.29 ± 0.09	39.0 ± 0.9	33 ± 1	0.4 ± 0.4	‐
Low	F	Without Obesity	40	111 ± 5	57 ± 2	0.8 ± 0.03^a,b^	0.88 ± 0.03	22.0 ± 1.7	20 ± 2	20 ± 1	1.10 ± 0.11	31.3 ± 1.1	38 ± 1	0.8 ± 0.5	8.3 ± 0.7
		With Obesity	40	115 ± 7	59 ± 4	0.95 ± 0.04	0.88 ± 0.05	26.2 ± 2.4	21 ± 3	22 ± 2	1.23 ± 0.16	31.3 ± 1.5	39 ± 2	1.4 ± 0.6	8.6 ± 1.0
	M	Without Obesity	60	104 ± 3	53 ± 2	1.07 ± 0.02	0.93 ± 0.02	26.6 ± 1.3	19 ± 1	18 ± 1	1.49 ± 0.08	26.8 ± 0.8	44 ± 1	1.9 ± 0.3	9.7 ± 0.5
		With Obesity	60	103 ± 4	54 ± 2	1.25 ± 0.02	0.94 ± 0.03	32.8 ± 1.5	22 ± 2	19 ± 1	1.89 ± 0.09	28.1 ± 0.9	42 ± 1	1.8 ± 0.4	8.8 ± 0.6
Mod	F	Without Obesity	60	133 ± 5	69 ± 2	1 ± 0.03^a,b^	0.93 ± 0.03	27.8 ± 1.7	26 ± 2	22 ± 1	1.31 ± 0.11	29.8 ± 1.1	39 ± 1	2.2 ± 0.5	10.3 ± 0.7
		With Obesity	60	132 ± 7	68 ± 4	1.16 ± 0.04	0.95 ± 0.05	33.0 ± 2.4	27 ± 3	22 ± 2	1.59 ± 0.16	30.1 ± 1.5	40 ± 2	2.4 ± 0.6	11.6 ± 1.0
	M	Without Obesity	80	121 ± 3	62 ± 2	1.31 ± 0.02	0.97 ± 0.02	33.9 ± 1.3	24 ± 1	22 ± 1	1.59 ± 0.08	26.8 ± 0.8	44 ± 1	2.7 ± 0.3	11.2 ± 0.5
		With Obesity	80	115 ± 4	60 ± 2	1.46 ± 0.02	0.96 ± 0.03	38.6 ± 1.5	26 ± 2	20 ± 1	2.04 ± 0.09	27.7 ± 0.9	42 ± 1	2.4 ± 0.4	10.1 ± 0.6
High	F	Without Obesity	80	155 ± 5 ^a^	80 ± 2 ^a^	1.23 ± 0.03^a,b^	0.99 ± 0.03	36.3 ± 1.7	34 ± 2	25 ± 1	1.48 ± 0.11	29.8 ± 1.1	39 ± 1	3.2 ± 0.5	11.7 ± 0.7
		With Obesity	80	148 ± 7	75 ± 4	1.39 ± 0.04	0.98 ± 0.05	40.7 ± 2.4	33 ± 3	24 ± 2	1.77 ± 0.16	29.8 ± 1.5	40 ± 2	4.6 ± 0.6	14.0 ± 1.0
	M	Without Obesity	100	140 ± 3	71 ± 2	1.59 ± 0.02	1.01 ± 0.02	44.5 ± 1.3	31 ± 1	26 ± 1	1.76 ± 0.08	27.5 ± 0.8	43 ± 1	3.3 ± 0.3	11.8 ± 0.5
		With Obesity	100	127 ± 4	67 ± 2	1.73 ± 0.02	1 ± 0.03	48.3 ± 1.5	33 ± 2	23 ± 1	2.17 ± 0.1	28 ± 0.9	41 ± 1	3.2 ± 0.4	11.1 ± 0.6
Interactions, *p* < 0.05*				Grp × WR, Sex × WR	Grp × WR, Sex × WR									Sex × WR	Sex × WR
Main Effects, *p* < 0.05**				Sex, WR	Sex, WR	Group, Sex, WR	WR	Group, Sex, WR	WR	WR	Group, Sex, WR	Sex, WR	Sex, WR	WR	WR

*Note*: *p < 0.05 for significant interactions (group × WR, sex × WR, group × sex × WR), post hoc differences are noted by: ^a^ *p* < 0.05 males vs. females within that WR; ^b^ *p* < 0.05 without vs. with obesity within that WR. **p < 0.05 for a significant main effect of group (with vs. without obesity), sex (male vs. female), and WR (rest, low, mod, high).

Abbreviations: %Pred HRmax, percent of predicted maximum heart rate; F, female; *f*_B_, breathing frequency; HR, heart rate; M, male; MVV, maximal voluntary ventilation; P_ET_CO_2_, partial pressure of end‐tidal carbon dioxide; RER, respiratory exchange ratio; RPB, ratings of perceived breathlessness; RPE, ratings of perceived exertion; V̇CO_2_, carbon dioxide production; V̇_E_, minute ventilation; V̇O_2_, oxygen update; V_T_, tidal volume; WR, work rate.

**TABLE 3:** Measures from the multidimensional dyspnea profile, each assessed on a scale of 1–10 and reported as mean ± SD.

	Female		Male		*p*‐value		
	Without obesity	With obesity	Without obesity	With obesity	Group × Sex	Group	Sex
*N*	10	5	18	14			
Unpleasantness	2.5 ± 1.4	4.0 ± 2.2	3.6 ± 2.3	3.2 ± 2.5	0.1938	0.5103	0.9541
Depressed	1.1 ± 0.3	2.2 ± 2.7	1.0 ± 0.0	1.1 ± 0.4	0.8929	0.1734	0.3584
Anxious	2.0 ± 1.6	3.0 ± 2.3	2.0 ± 1.7	2.1 ± 1.4	0.7463	0.3974	0.5728
Frustrated	1.7 ± 1.9	3.8 ± 2.3	2.3 ± 2.2	1.5 ± 1.3	0.0030	0.2013	0.1783
Angry	1.3 ± 0.9	2.2 ± 1.8	1.2 ± 0.4	1.0 ± 0.0	0.0310	0.4481	0.0848
Afraid	1.2 ± 0.4	1.4 ± 0.5	1.2 ± 0.5	1.1 ± 0.4	0.4918	0.3915	0.1968
My breath does not go in all the way	3.3 ± 2.1	4.4 ± 3.0	2.5 ± 1.5	3.0 ± 2.3	0.7804	0.4355	0.1779
My breathing requires effort	4.3 ± 3.1	3.2 ± 2.7	3.2 ± 2.1	3.1 ± 1.9	0.5672	0.5227	0.7082
I feel that I am smothering	1.4 ± 1.0	3.0 ± 3.5	1.9 ± 1.3	2.0 ± 2.0	0.2938	0.5480	0.7025
I feel hunger for air	1.8 ± 1.1	2.8 ± 2.5	2.0 ± 1.4	1.9 ± 1.4	0.5167	0.5344	0.6608
My breathing is heavy	3.8 ± 2.3	4.0 ± 2.3	3.4 ± 2.4	3.1 ± 2.2	0.7579	0.9213	0.3089
I feel out of breath	3.7 ± 2.5	2.6 ± 1.7	2.9 ± 1.8	2.3 ± 1.7	0.8394	0.2325	0.4241
My chest feels tight	3.0 ± 2.7	3.2 ± 2.2	2.3 ± 1.9	1.9 ± 1.1	0.5375	0.5501	0.2880
My breathing requires work	3.5 ± 2.8	3.0 ± 2.3	2.8 ± 2.1	2.8 ± 1.9	0.8796	0.8388	0.7459
I feel that I am suffocating	1.1 ± 0.3	2.8 ± 3.0	1.4 ± 0.9	1.2 ± 0.4	0.1147	0.3117	0.7777
My chest is constricted	2.3 ± 1.8	2.2 ± 1.6	2.1 ± 1.7	1.4 ± 1.1	0.3863	0.5292	0.2085
I feel that my breathing is rapid	2.8 ± 1.5	2.2 ± 1.8	2.7 ± 1.6	2.7 ± 1.9	0.5282	0.3379	0.7231
My breathing is shallow	3.4 ± 2.9	3.2 ± 2.9	1.9 ± 1.2	2.1 ± 1.6	0.8510	0.8053	0.1008
I feel that I am breathing more	3.5 ± 2.2	5.2 ± 3.4	3.8 ± 2.0	3.4 ± 2.0	0.2268	0.5590	0.4342
I cannot get enough air	2.3 ± 1.6	2.8 ± 1.8	2.6 ± 1.8	1.8 ± 1.6	0.2762	0.7962	0.4195
My breath does not go out all the way	3.7 ± 2.8	3.4 ± 1.7	2.4 ± 1.7	1.9 ± 1.6	0.3781	0.6259	0.0321

### 3.3 Operating lung volumes and ventilatory constraints

**FIGURE 1:**
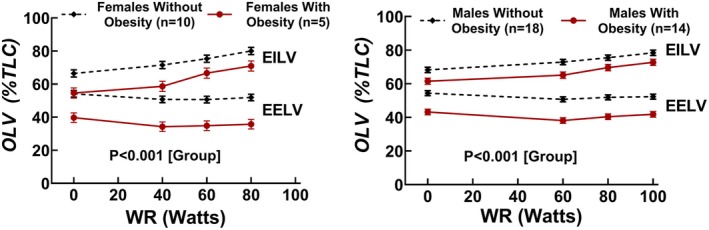
Operating lung volumes among participants with and without obesity by sex (left panel: females and right panel: males). OLV, operating lung volume; TLC, total lung capacity; EILV, end‐inspiratory lung volume; EELV, end‐expiratory lung volume.

Individuals with obesity experienced lower operating lung volumes during exercise compared with those without obesity (*p* < 0.0001 for EELV and EILV; Figure 1). EFL was observed in 16%, 16%, and 21% of individuals with obesity during low, moderate, and higher work rates, respectively, compared with 0%, 0%, and 4% of individuals without obesity; these differences did not achieve statistical significance (*p* = 0.0598, *p* = 0.0598, and *p* = 0.1424, respectively). There were no significant sex differences in the prevalence of EFL during exercise. Dynamic hyperinflation was observed in 5%, 16%, and 26% of individuals with obesity at low, moderate, and higher work rates, respectively, compared with 0%, 7%, and 7% of individuals without obesity; these differences also did not achieve statistical significance (*p* = 0.4043, *p* = 0.3712, and *p* = 0.1016, respectively). 22% of males experienced dynamic hyperinflation during the higher level of exercise compared with 0% of females (*p* = 0.0799).

### 3.4 Exertional symptoms

**FIGURE 2:**
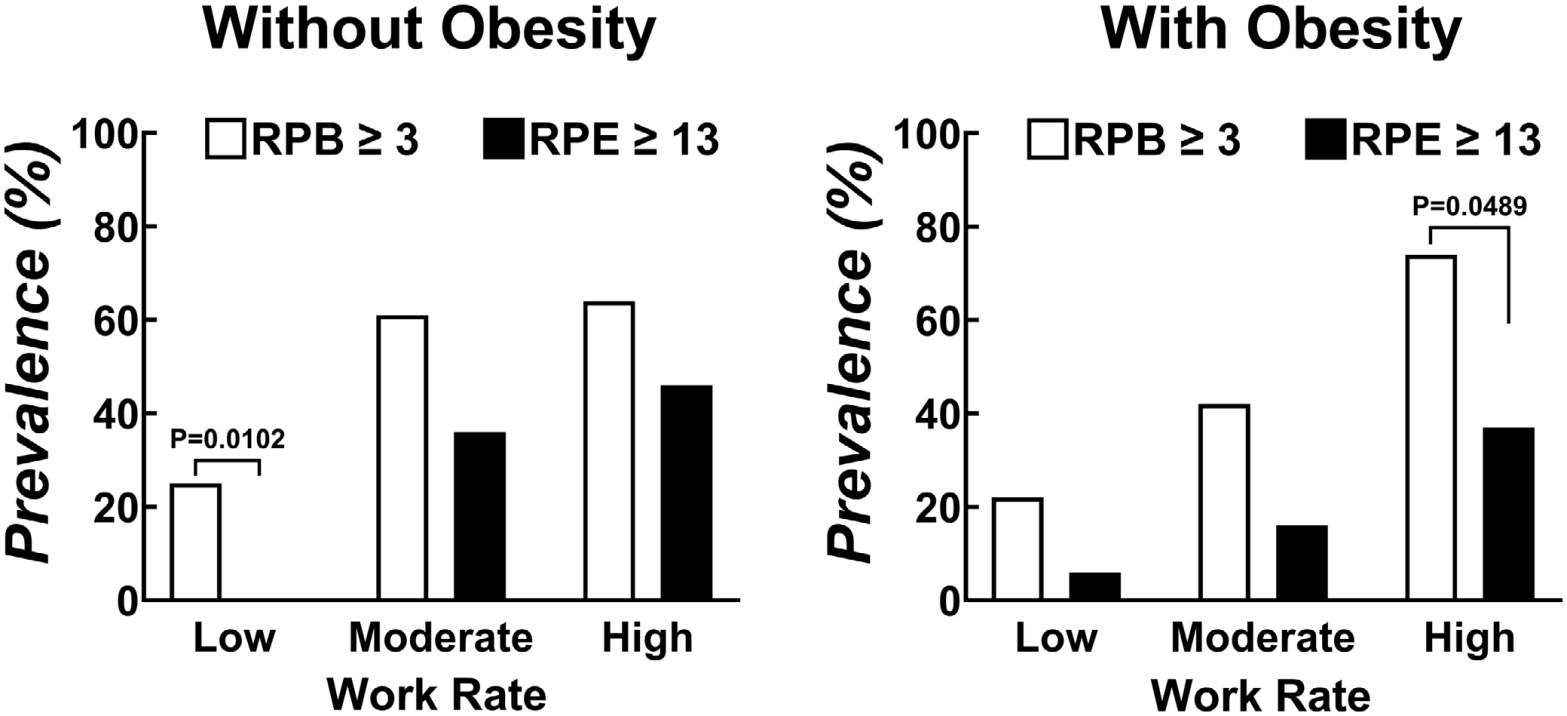
Proportions of participants rating moderate or strong breathlessness (RPB ≥3) during exercise and somewhat hard or greater level of whole‐body exertion (RPE ≥13) at the low, moderate, and higher work rates. Results are shown for all participants, those without obesity (*n* = 28; left panel) and those with obesity (*n* = 19; right panel). RPB, rate of perceived breathlessness; RPE, rate of perceived exertion.

74% of individuals with obesity and 64% of individuals without obesity reported moderate or greater breathlessness (i.e., RPB ≥3) at the higher work rate (i.e., 80 W for females, 100 W for males). Individuals with obesity were twice as likely to report moderate or greater breathlessness than to rate their whole‐body exertion as somewhat hard or greater (RPE ≥13) at higher work rate (Odds Ratio: 2.00; 95% CI 1.10 to 4.00; *p* = 0.0489) (Figure 2). Females with obesity reported greater feelings of frustration accompanying their breathlessness compared with males with obesity (*p* < 0.05; Table 3). Females also reported higher ratings for the descriptor “my breath does not go out all the way” compared with males (*p* = 0.0321; Table 3). There were no other significant group or sex differences in negative emotions or descriptors of breathlessness related to exercise.

## 4 DISCUSSION

This is the first investigation on the effects of obesity on pulmonary function, ventilatory constraints, and exertional breathlessness among e‐cigarette users. We found that individuals with obesity reported greater e‐cigarette use when compared with those without obesity, and individuals with obesity demonstrated lower operating lung volumes and tended to experience greater ventilatory constraints during exercise compared to those without obesity. Despite these differences, exertional breathlessness was not different between e‐cigarette users with and without obesity. Perceived breathlessness ratings for all e‐cigarette users were disproportionately higher than perceived exertion ratings at similar levels of exercise, and most participants reported moderate or strong exertional breathlessness during relatively moderate intensity exercise. Finally, pulmonary function (FVC, FEV_1_, and diffusion capacity) was largely well‐preserved among e‐cigarette users, but mid‐expiratory flows were below 80% of predicted for 34% of participants; this relatively high prevalence of reduced mid‐expiratory flows could indicate small airway dysfunction despite preserved FEV_1_ and FVC. There were no differences in mid‐expiratory flows between individuals with and without obesity.

Excess weight on the chest wall and abdomen creates conditions for low lung volume breathing among otherwise healthy individuals with obesity, where expiratory flow and volume reserves are limited (Babb, 2013a). Obesity is also associated with increased metabolic and ventilatory demands during exercise and greater mechanical ventilatory constraints such as EFL and dynamic hyperinflation, which could contribute to exertional breathlessness and exercise intolerance (Babb, 2013b; Jensen et al., 2009; Sin et al., 2002). In the current study, individuals with obesity who use e‐cigarettes had lower operating lung volumes and were more likely to experience EFL during exercise compared with those without obesity. Participants with obesity were also more likely to experience dynamic hyperinflation, although this finding did not reach statistical significance. Obesity was also associated with more e‐cigarette puffs taken each day, which is consistent with a greater likelihood of regular e‐cigarette use among individuals with obesity (Delk et al., 2018; Lanza et al., 2017). A potential explanation for the increased use of e‐cigarettes in individuals with obesity could include their use as a weight loss or weight control strategy (Morean & Wedel, 2017), although more research is needed in this space to better understand reasons for increased e‐cigarette use behaviors among individuals with obesity.

Individuals with obesity reportedly experience greater exertional breathlessness compared with those without obesity (Goh et al., 2023; Hagenburg et al., 2022). 52% and 67% of all participants experienced significant breathlessness (RPB ≥3) during the moderate and higher work rates, respectively. Three out of four individuals with obesity experienced significant breathlessness at the higher work rate. These findings are consistent with a growing body of literature linking e‐cigarette use with increased likelihood of respiratory symptoms in asthmatic adolescent e‐cigarette users vs. never smokers (Wills et al., 2021), and among otherwise healthy young adults (Chaffee et al., 2021). Most prior studies have relied on retrospective symptom reporting in lieu of assessments during physiological stress (i.e., during exercise). Our results are consistent with a recent cross‐sectional study among e‐cigarette users with at least 2 years of daily use (*n* = 20), which found higher exertional breathlessness ratings during exercise when compared with age‐, height‐ and sex‐matched controls (*n* = 20), despite normal lung function (Williams et al., 2026). The authors also reported reduced pulmonary vascular responsiveness among e‐cigarette users compared with controls. These data along other reports of gas exchange abnormalities, ventilation inhomogeneity and pulmonary inflammation in e‐cigarette users offer a mechanistic basis for increased respiratory symptoms (Puliyakote et al., 2021; Stanojevic et al., 2025; Wetherill et al., 2023); although direct comparisons between e‐cigarette users with and without obesity are missing from the literature. Together, these data reinforce the importance of assessing symptoms under physiological stress where subtle abnormalities in gas‐exchange may become clinically apparent.

Prior cross‐sectional studies have reported reductions in spirometric indices such as FEV_1_, mid‐expiratory flows, and FEV_1_/FVC ratio among e‐cigarette users relative to healthy non‐users, indicative of increased airway resistance and airflow obstruction (Jiang et al., 2022; Darabseh et al., 2021; Joshi et al., 2021; Meo et al., 2018). In the present study, spirometric indices appeared relatively well preserved among participants with and without obesity. However, since our study did not include a healthy non‐user group, we are unable to determine whether spirometry indices in our e‐cigarette users were reduced relative to a healthy matched group of young adults. Approximately one‐third of participants in our study (39% of those without obesity and 26% of those with obesity) exhibited lower mid‐expiratory flows, which may indicate small airway dysfunction. These findings could be clinically relevant because small airway dysfunction is increasingly being recognized as an early marker in the development of chronic lung disease (Zhou et al., 2025).

There are well‐established sex differences in lung function and exercise responses (Bhammar et al., 2022), which in addition to differing work rates for males and females, necessitated reporting results by sex. There were no sex differences in the intensity of exertional breathlessness, which is consistent with a previous study among individuals with obesity who were not e‐cigarette users (Bhammar et al., 2022) but contrasts with epidemiological studies that report higher odds of breathlessness among females compared with males (Ekström et al., 2018; Lamprecht et al., 2013). These differences could be related to methodology, as epidemiological studies typically rely on self‐reported retrospective single‐item breathlessness surveys whereas applied physiological studies assess RPB in real time during exercise.

Beyond the intensity of breathlessness, qualitative descriptors and emotional responses can provide insights into the perceptual experience of breathlessness (Parshall et al., 2012). Compared with males with obesity, females with obesity reported greater feelings of frustration and anger, and provided higher ratings for the descriptor “my breath does not go out all the way.” These differences in the qualitative aspects of breathlessness could reflect underlying psychophysiological sex differences. Females have lower absolute lung volumes, narrower airways, and less respiratory musculature, which leads to increased work of breathing and could lead to feelings of incomplete expiration and overall breathing discomfort (Bhammar et al., 2022; Schaeffer et al., 2014). Supporting this interpretation, research in patients who completed a multidimensional dyspnea profile anchored to their most severe episode of breathlessness within the past 6 weeks suggests that despite similar intensity ratings for breathlessness between males and females, females report greater feelings of breathing discomfort, muscle work or effort, and chest tightness as well as increased frustration and anxiety in response to breathlessness (Milne et al., 2025). Furthermore, consistent with our findings, 50% of females selected the descriptor “my breath does not go out all the way” in that study (Milne et al., 2025). These results highlight the importance of multidimensional assessments of breathlessness since intensity measures alone may fail to capture the emotional and affective impact of breathlessness, which has implications for initiating and sustaining exercise and the long‐term engagement of physical activity.

There are several limitations to be acknowledged. The absence of a non‐user control group limits our ability to draw any conclusions regarding the interaction between e‐cigarette use and obesity on pulmonary function, respiratory symptoms, and ventilatory constraints during exercise. We also did not exclude participants with a smoking history. Cigarettes were the first tobacco product used for 51% of participants, with an initiation age of 15.1 ± 3.2 years, and half reporting prior regular use (11 males and 1 female). Details regarding smoking history (i.e., pack years) were not available, making it difficult to understand whether smoking may have affected study outcomes. However, current smokers were excluded from participation, which was verified by exhaled carbon monoxide (Benowitz et al., 2020). While sex‐specific analyses were conducted, the relatively small sample of females with obesity (*n* = 5) limited statistical power and may have reduced our ability to detect meaningful differences involving this subgroup. Finally, this study was not designed to evaluate the effects of e‐cigarette use on cardiorespiratory fitness, which is why we chose submaximal exercise testing in lieu of a maximal exercise test to volitional exhaustion. Three previous studies have reported lower cardiorespiratory fitness among e‐cigarette users compared with non‐user controls (Simovic et al., 2024; Tattersall et al., 2023; Williams et al., 2026), though the effect of obesity status on cardiorespiratory fitness among e‐cigarette users remains unclear. Despite these limitations, we have provided a comprehensive characterization of pulmonary function, cardiorespiratory responses, and exertional symptoms in e‐cigarette users with and without obesity, addressing a notable gap in the literature regarding the potential health effects of obesity among e‐cigarette users.

In conclusion, individuals with obesity report greater e‐cigarette use behaviors and potentially experience greater ventilatory constraints during exercise compared with those without obesity. At relatively high work rates, e‐cigarette users with obesity experience exertional breathlessness that is out of proportion to their feelings of whole‐body exertion. Future studies with larger samples and non‐user control groups are needed to better understand physiological mechanisms underlying respiratory symptoms and to determine whether early physiological abnormalities in pulmonary function contribute to long‐term declines in physical activity and cardiorespiratory fitness in this population.

## AUTHOR CONTRIBUTIONS

Dharini M. Bhammar conceived and designed the study. Funding for the study was obtained by Dharini M. Bhammar and Theodore L. Wagener. Dharini M. Bhammar, Theodore L. Wagener, and Alice Hinton developed the methodology. Dharini M. Bhammar, Joseph W. Walsh, and Michael Beck conducted the investigation. Dharini M. Bhammar, Michael Beck, and Joseph W. Walsh curated the data, and Dharini M. Bhammar and Alice Hinton performed the formal analyses. Dharini M. Bhammar administered the project, secured study resources, supervised the research, and validated the findings. Dharini M. Bhammar and Joseph W. Walsh contributed to data visualization. Joseph W. Walsh and Dharini M. Bhammar drafted the original manuscript. All authors (Joseph W. Walsh, Michael Beck, Alice Hinton, Theodore L. Wagener, Jason E. Lang, Jing G. Wang, and Dharini M. Bhammar) reviewed and edited the manuscript and approved the final version.

## FUNDING INFORMATION

This work was supported by an OSU Division of Medical Oncology Grant (Dharini M. Bhammar and Theodore L. Wagener); National Institutes of Health (NIH) grant no. K12CA133250 (Dharini M. Bhammar); and the National Center for Advancing Translational Sciences grant no. UM1TR004548.

## CONFLICT OF INTEREST STATEMENT

The authors have no relevant conflicts of interest to disclose. The results of this study are presented clearly, honestly, and without fabrication, falsification, or inappropriate data manipulation.

## ETHICS STATEMENT

The study was conducted according to the guidelines laid down by the Declaration of Helsinki and all procedures were approved by the The Ohio State University Cancer Institutional Review Board (approval no. 2022C0202). All participants provided written, informed consent.

## Data Availability

Data will be made available upon reasonable request.

## References

[phy270993-bib-0001] Puliyakote, A. S. K. , Elliott, A. R. , Sa, R. C. , Anderson, K. M. , Alexander, L. E. C. , & Hopkins, S. R. (2021). Vaping disrupts ventilation‐perfusion matching in asymptomatic users. Journal of Applied Physiology, 130, 308–317. 33180648 10.1152/japplphysiol.00709.2020 PMC7948111

[phy270993-bib-0002] Babb, T. G. (2013a). Exercise ventilatory limitation: the role of expiratory flow limitation. Exercise and Sport Sciences Reviews, 41, 11–18. 23038244 10.1097/JES.0b013e318267c0d2 PMC3529766

[phy270993-bib-0003] Babb, T. G. (2013b). Obesity: challenges to ventilatory control during exercise‐‐a brief review. Respiratory Physiology & Neurobiology, 189, 364–370. 23707540 10.1016/j.resp.2013.05.019 PMC3797147

[phy270993-bib-0004] Babb, T. G. , Ranasinghe, K. G. , Comeau, L. A. , Semon, T. L. , & Schwartz, B. (2008). Dyspnea on exertion in obese women: association with an increased oxygen cost of breathing. American Journal of Respiratory and Critical Care Medicine, 178, 116–123. 18420968 10.1164/rccm.200706-875OC

[phy270993-bib-0005] Balmain, B. N. , Weinstein, K. , Bernhardt, V. , Marines‐Price, R. , Tomlinson, A. R. , & Babb, T. G. (2020). Multidimensional aspects of dyspnea in obese patients referred for cardiopulmonary exercise testing. Respiratory Physiology & Neurobiology, 274, 103365. 31899350 10.1016/j.resp.2019.103365 PMC7002243

[phy270993-bib-0006] Bames, J. , Behrens, T. K. , Benden, M. E. , Biddle, S. , Bond, D. , Brassard, P. , Brown, H. , Carr, L. , Carson, V. , & Chaput, J. (2012). Standardized use of the terms “sedentary” and “sedentary behaviours”. Applied Physiology, Nutrition, and Metabolism = Physiologie Appliquee, Nutrition Et Metabolisme, 37, 540–542. 22540258 10.1139/h2012-024

[phy270993-bib-0007] Banzett, R. B. , O'Donnell, C. R. , Guilfoyle, T. E. , Parshall, M. B. , Schwartzstein, R. M. , Meek, P. M. , Gracely, R. H. , & Lansing, R. W. (2015). Multidimensional Dyspnea Profile: an instrument for clinical and laboratory research. European Respiratory Journal, 45, 1681–1691. 25792641 10.1183/09031936.00038914 PMC4450151

[phy270993-bib-0008] Benowitz, N. L. , Bernert, J. T. , Foulds, J. , Hecht, S. S. , Jacob, P. , Jarvis, M. J. , Joseph, A. , Oncken, C. , & Piper, M. E. (2020). Biochemical Verification of Tobacco Use and Abstinence: 2019 Update. Nicotine and Tobacco Research, 22, 1086–1097. 31570931 10.1093/ntr/ntz132 PMC7882145

[phy270993-bib-0009] Bernhardt, V. , & Babb, T. G. (2014). Weight loss reduces dyspnea on exertion in obese women. Respiratory Physiology & Neurobiology, 204, 86–92. 25220695 10.1016/j.resp.2014.09.004 PMC4254018

[phy270993-bib-0010] Bernhardt, V. , Bhammar, D. M. , Marines‐Price, R. , & Babb, T. G. (2019). Weight loss reduces dyspnea on exertion and unpleasantness of dyspnea in obese men. Respiratory Physiology & Neurobiology, 261, 55–61. 30658095 10.1016/j.resp.2019.01.007 PMC6368458

[phy270993-bib-0011] Bernhardt, V. , Stickford, J. L. , Bhammar, D. M. , Balmain, B. N. , & Babb, T. G. (2022). Repeatability of dyspnea measurements during exercise in women with obesity. Respiratory Physiology & Neurobiology, 297, 103831. 34922000 10.1016/j.resp.2021.103831 PMC11463220

[phy270993-bib-0012] Bhammar, D. M. , Balmain, B. N. , Babb, T. G. , & Bernhardt, V. (2022). Sex differences in the ventilatory responses to exercise in mild to moderate obesity. Experimental Physiology, 107, 965–977. 35771362 10.1113/EP090309 PMC9357174

[phy270993-bib-0013] Bhammar, D. M. , Stickford, J. L. , Bernhardt, V. , & Babb, T. G. (2016). Effect of weight loss on operational lung volumes and oxygen cost of breathing in obese women. International Journal of Obesity, 40, 998–1004. 26869243 10.1038/ijo.2016.21 PMC4899150

[phy270993-bib-0014] Borg, G. (1970). Perceived exertion as an indicator of somatic stress. Scandinavian Journal of Rehabilitation Medicine, 2, 92–98. 5523831

[phy270993-bib-0015] Borg, G. A. (1982). Psychophysical bases of perceived exertion. Medicine and Science in Sports and Exercise, 14, 377–381. 7154893

[phy270993-bib-0016] Burrows, B. , Kasik, J. E. , Niden, A. H. , & Barclay, W. R. (1961). Clinical usefulness of the single‐breath pulmonary diffusing capacity test. American Review of Respiratory Disease, 84, 789–806. 13875021 10.1164/arrd.1961.84.6.789

[phy270993-bib-0017] Chaffee, B. W. , Barrington‐Trimis, J. , Liu, F. , Wu, R. , McConnell, R. , Krishnan‐Sarin, S. , Leventhal, A. M. , & Kong, G. (2021). E‐cigarette use and adverse respiratory symptoms among adolescents and young adults in the United States. Preventive Medicine, 153, 106766. 34418439 10.1016/j.ypmed.2021.106766 PMC8595821

[phy270993-bib-0018] Conover, W. J. , & Iman, R. L. (1981). Rank transformations as a bridge between parametric and nonparametric statistics. The American Statistician, 35, 124–129.

[phy270993-bib-0019] Cooper, B. G. , Stocks, J. , Hall, G. L. , Culver, B. , Steenbruggen, I. , Carter, K. W. , Thompson, B. R. , Graham, B. L. , Miller, M. R. , Ruppel, G. , Henderson, J. , Vaz Fragoso, C. A. , & Stanojevic, S. (2017). The Global lung function initiative (GLI) Network: bringing the world's respiratory reference values together. Breathe (Sheffield, England), 13, e56–e64. 28955406 10.1183/20734735.012717 PMC5607614

[phy270993-bib-0020] Darabseh, M. Z. , Selfe, J. , Morse, C. I. , & Degens, H. (2021). Impact of vaping and smoking on maximum respiratory pressures and respiratory function. International Journal of Adolescence and Youth, 26, 421–431.

[phy270993-bib-0021] Delk, J. , Creamer, M. R. , Perry, C. L. , & Harrell, M. B. (2018). Weight status and cigarette and electronic cigarette use in adolescents. American Journal of Preventive Medicine, 54, e31–e35. 29132954 10.1016/j.amepre.2017.09.007 PMC5736419

[phy270993-bib-0022] Ekström, M. , Sundh, J. , Schiöler, L. , Lindberg, E. , Rosengren, A. , Bergström, G. , Angerås, O. , Hedner, J. , Brandberg, J. , & Bake, B. (2018). Absolute lung size and the sex difference in breathlessness in the general population. PLoS One, 13, e0190876. 29304074 10.1371/journal.pone.0190876 PMC5755925

[phy270993-bib-0023] Gilman, S. A. , & Banzett, R. B. (2009). Physiologic changes and clinical correlates of advanced dyspnea. Current Opinion in Supportive and Palliative Care, 3, 93–97. 19421065 10.1097/SPC.0b013e32832b42ba PMC2834174

[phy270993-bib-0024] Goh, J. T. , Balmain, B. N. , Wilhite, D. P. , Granados, J. , Sandy, L. L. , Liu, Y.‐L. , Pawelczyk, J. A. , & Babb, T. G. (2023). Elevated risk of dyspnea in adults with obesity. Respiratory Physiology & Neurobiology, 318, 104151. 37673304 10.1016/j.resp.2023.104151 PMC11087888

[phy270993-bib-0025] Goldman, H. I. , & Becklake, M. R. (1959). Respiratory Function Tests‐Normal Values at Median Altitudes and the Prediction of Normal Results. American Review of Tuberculosis and Pulmonary Diseases, 79, 457–467. 10.1164/artpd.1959.79.4.457 13650117

[phy270993-bib-0026] Hagenburg, J. , Bertin, E. , Salmon, J.‐H. , Thierry, A. , Perotin, J.‐M. , Dormoy, V. , Dury, S. , Gaubil, I. , Bolko, L. , & Lebargy, F. (2022). Association between obesity‐related dyspnea in daily living, lung function and body composition analyzed by DXA: a prospective study of 130 patients. BMC Pulmonary Medicine, 22, 103. 35337302 10.1186/s12890-022-01884-5 PMC8957162

[phy270993-bib-0027] Harris, P. A. , Taylor, R. , Minor, B. L. , Elliott, V. , Fernandez, M. , O'Neal, L. , McLeod, L. , Delacqua, G. , Delacqua, F. , Kirby, J. , & Duda, S. N. (2019). The REDCap consortium: Building an international community of software platform partners. Journal of Biomedical Informatics, 95, 103208. 31078660 10.1016/j.jbi.2019.103208 PMC7254481

[phy270993-bib-0028] Harris, P. A. , Taylor, R. , Thielke, R. , Payne, J. , Gonzalez, N. , & Conde, J. G. (2009). Research electronic data capture (REDCap)—A metadata‐driven methodology and workflow process for providing translational research informatics support. Journal of Biomedical Informatics, 42, 377–381. 18929686 10.1016/j.jbi.2008.08.010 PMC2700030

[phy270993-bib-0029] Jensen, D. , Ofir, D. , & O'Donnell, D. E. (2009). Effects of pregnancy, obesity and aging on the intensity of perceived breathlessness during exercise in healthy humans. Respiratory Physiology & Neurobiology, 167, 87–100. 19450766 10.1016/j.resp.2009.01.011

[phy270993-bib-0030] Jiang, E. , Vieira Pires, N. , Hawkins, D. , Sangapalaarachchi, D. , Ilievski, V. , Loiacono, N. , Navas Acien, A. , & Oelsner, E. (2022). E‐cigarette use and lung function: preliminary results from the VapeScan Study. European Respiratory Journal, 60, 4638.

[phy270993-bib-0031] Joshi, D. , Duong, M. , Kirkland, S. , & Raina, P. (2021). Impact of electronic cigarette ever use on lung function in adults aged 45‐85: a cross‐sectional analysis from the Canadian Longitudinal Study on Aging. BMJ Open, 11, e051519. 10.1136/bmjopen-2021-051519 PMC8552144 34706955

[phy270993-bib-0032] Lamprecht, B. , Vanfleteren, L. E. , Studnicka, M. , Allison, M. , MA, M. B. , Vollmer, W. M. , Tan, W. C. , Nielsen, R. , Nastalek, P. , Gnatiuc, L. , Kaiser, B. , Janson, C. , Wouters, E. F. , Burney, P. , Buist, A. S. , & Group BCR . (2013). Sex‐related differences in respiratory symptoms: results from the BOLD Study. European Respiratory Journal, 42, 858–860. 24000253 10.1183/09031936.00047613 PMC3759301

[phy270993-bib-0033] Lanza, H. I. , Pittman, P. , & Batshoun, J. (2017). Obesity and cigarette smoking: Extending the link to e‐cigarette/vaping use. American Journal of Health Behavior, 41, 338–347. 28376978 10.5993/AJHB.41.3.13 PMC5506838

[phy270993-bib-0034] Macintyre, N. , Crapo, R. O. , Viegi, G. , Johnson, D. C. , van der Grinten, C. P. , Brusasco, V. , Burgos, F. , Casaburi, R. , Coates, A. , Enright, P. , Gustafsson, P. , Hankinson, J. , Jensen, R. , McKay, R. , Miller, M. R. , Navajas, D. , Pedersen, O. F. , Pellegrino, R. , & Wanger, J. (2005). Standardisation of the single‐breath determination of carbon monoxide uptake in the lung. European Respiratory Journal, 26, 720–735. 16204605 10.1183/09031936.05.00034905

[phy270993-bib-0035] Mahler, D. A. , Harver, A. , Lentine, T. , Scott, J. A. , Beck, K. , & Schwartzstein, R. M. (1996). Descriptors of breathlessness in cardiorespiratory diseases. American Journal of Respiratory and Critical Care Medicine, 154, 1357–1363. 8912748 10.1164/ajrccm.154.5.8912748

[phy270993-bib-0036] Meo, S. A. , Ansary, M. A. , Barayan, F. R. , Almusallam, A. S. , Almehaid, A. M. , Alarifi, N. S. , Alsohaibani, T. A. , & Zia, I. (2018). Electronic Cigarettes: Impact on Lung Function and Fractional Exhaled Nitric Oxide Among Healthy Adults. American Journal of Men's Health, 13, 1557988318806073. 10.1177/1557988318806073 PMC6771130 30318975

[phy270993-bib-0037] Milic‐Emili, J. (2000). Expiratory flow limitation ‐ Detection and clinical implications ‐ Roger S. Mitchell Lecture. Chest, 117, 219s–223s. 10.1378/chest.117.5_suppl_1.219s-a 10843918

[phy270993-bib-0038] Miller, M. R. , Hankinson, J. , Brusasco, V. , Burgos, F. , Casaburi, R. , Coates, A. , Crapo, R. , Enright, P. , van der Grinten, C. P. , Gustafsson, P. , Jensen, R. , Johnson, D. C. , MacIntyre, N. , McKay, R. , Navajas, D. , Pedersen, O. F. , Pellegrino, R. , Viegi, G. , Wanger, J. , & Force, A. E. T. (2005). Standardisation of spirometry. European Respiratory Journal, 26, 319–338. 16055882 10.1183/09031936.05.00034805

[phy270993-bib-0039] Milne, K. M. , Zhang, J. , Harris, O. D. , & Guenette, J. A. (2025). Sex‐differences in the multidimensional evaluation of dyspnea in respiratory outpatients. Frontiers in Medicine, 12, 1627496. 40703296 10.3389/fmed.2025.1627496 PMC12283695

[phy270993-bib-0040] Morean, M. E. , & Wedel, A. V. (2017). Vaping to lose weight: Predictors of adult e‐cigarette use for weight loss or control. Addictive Behaviors, 66, 55–59. 27875790 10.1016/j.addbeh.2016.10.022

[phy270993-bib-0041] O'Donnell, D. E. , O'Donnell, C. D. , Webb, K. A. , & Guenette, J. A. (2012). Respiratory Consequences of Mild‐to‐Moderate Obesity: Impact on Exercise Performance in Health and in Chronic Obstructive Pulmonary Disease. Pulmonary Medicine, 2012, 818925. 23097698 10.1155/2012/818925 PMC3477561

[phy270993-bib-0042] O'Donnell, D. E. , Revill, S. M. , & Webb, K. A. (2001). Dynamic hyperinflation and exercise intolerance in chronic obstructive pulmonary disease. American Journal of Respiratory and Critical Care Medicine, 164, 770–777. 11549531 10.1164/ajrccm.164.5.2012122

[phy270993-bib-0043] Parshall, M. B. , Schwartzstein, R. M. , Adams, L. , Banzett, R. B. , Manning, H. L. , Bourbeau, J. , Calverley, P. M. , Gift, A. G. , Harver, A. , Lareau, S. C. , Mahler, D. A. , Meek, P. M. , O'Donnell, D. E. , & American Thoracic Society Committee on D . (2012). An official American Thoracic Society statement: update on the mechanisms, assessment, and management of dyspnea. American Journal of Respiratory and Critical Care Medicine, 185, 435–452. 22336677 10.1164/rccm.201111-2042ST PMC5448624

[phy270993-bib-0044] Schaeffer, M. R. , Mendonca, C. T. , Levangie, M. C. , Andersen, R. E. , Taivassalo, T. , & Jensen, D. (2014). Physiological mechanisms of sex differences in exertional dyspnoea: role of neural respiratory motor drive. Experimental Physiology, 99, 427–441. 24213856 10.1113/expphysiol.2013.074880

[phy270993-bib-0045] Simovic, T. , Matheson, C. , Cobb, K. , Heefner, A. , Thode, C. , Colon, M. , Tunon, E. , Billingsley, H. , Salmons, H. , Ahmed, S. I. , Carbone, S. , Garten, R. , Breland, A. , Cobb, C. O. , Nana‐Sinkam, P. , & Rodriguez‐Miguelez, P. (2024). Young users of electronic cigarettes exhibit reduced cardiorespiratory fitness. Journal of Applied Physiology, 137, 569–580. 38990977 10.1152/japplphysiol.00292.2024 PMC11424176

[phy270993-bib-0046] Sin, D. D. , Jones, R. L. , & Man, S. F. (2002). Obesity is a risk factor for dyspnea but not for airflow obstruction. Archives of Internal Medicine, 162, 1477–1481. 12090884 10.1001/archinte.162.13.1477

[phy270993-bib-0047] Stanojevic, S. , Yung, M. H. , Sahin, B. , Johnson, N. , Stewart, H. , Laflamme, O. D. , Maksym, G. , Mateos‐Corral, D. , & Asbridge, M. (2025). Association between e‐cigarette exposure and ventilation homogeneity in young adults: a cross‐sectional study. European Respiratory Journal, 65, 2401675. 39603668 10.1183/13993003.01675-2024 PMC11948420

[phy270993-bib-0048] Strozza, D. , Wilhite, D. P. , Babb, T. G. , & Bhammar, D. M. (2020). Pitfalls in Expiratory Flow Limitation Assessment at Peak Exercise in Children: Role of Thoracic Gas Compression. Medicine and Science in Sports and Exercise, 52, 2310–2319. 33064406 10.1249/MSS.0000000000002378 PMC7573195

[phy270993-bib-0049] Tackett, A. P. , Urman, R. , Barrington‐Trimis, J. , Liu, F. , Hong, H. , Pentz, M. A. , Islam, T. S. , Eckel, S. P. , Rebuli, M. , & Leventhal, A. (2024). Prospective study of e‐cigarette use and respiratory symptoms in adolescents and young adults. Thorax, 79, 163–168. 37582630 10.1136/thorax-2022-218670 PMC11062480

[phy270993-bib-0050] Tattersall, M. C. , Hughey, C. M. , Piasecki, T. M. , Korcarz, C. E. , Hansen, K. M. , Ott, N. R. , Sandbo, N. , Fiore, M. C. , Baker, T. B. , & Stein, J. H. (2023). Cardiovascular and Pulmonary Responses to Acute Use of Electronic Nicotine Delivery Systems and Combustible Cigarettes in Long‐Term Users. Chest, 164, 757–769. 37044158 10.1016/j.chest.2023.03.047 PMC10504598

[phy270993-bib-0051] Vahratian, A. , Briones, E. M. , Jamal, A. , & Marynak, K. L. (2025). Electronic cigarette use among adults in the United States, 2019–2023. NCHS Data Briefs National Center for Health Statistics (US). 10.15620/cdc/174583 PMC12035660 40036117

[phy270993-bib-0052] Wanger, J. , Clausen, J. L. , Coates, A. , Pedersen, O. F. , Brusasco, V. , Burgos, F. , Casaburi, R. , Crapo, R. , Enright, P. , van der Grinten, C. P. , Gustafsson, P. , Hankinson, J. , Jensen, R. , Johnson, D. , Macintyre, N. , McKay, R. , Miller, M. R. , Navajas, D. , Pellegrino, R. , & Viegi, G. (2005). Standardisation of the measurement of lung volumes. European Respiratory Journal, 26, 511–522. 16135736 10.1183/09031936.05.00035005

[phy270993-bib-0053] Wetherill, R. R. , Doot, R. K. , Young, A. J. , Lee, H. , Schubert, E. K. , Wiers, C. E. , Leone, F. T. , Mach, R. H. , Kranzler, H. R. , & Dubroff, J. G. (2023). Molecular imaging of pulmonary inflammation in electronic and combustible cigarette users: a pilot study. Journal of Nuclear Medicine, 122, 264529. 10.2967/jnumed.122.264529 PMC10152129 36657981

[phy270993-bib-0054] Williams, T. G. , Collins, S. , Brotto, A. R. , D'Souza, A. W. , Ehnes, C. M. , Hicks, B. , Weatherald, J. , Leung, J. M. , & Stickland, M. K. (2026). Young chronic e‐cigarette users display cardiopulmonary abnormalities during exercise and blunted recruitment of pulmonary diffusing capacity. Chest, 169(6), 1616–1627. 41485699 10.1016/j.chest.2025.12.024 PMC13269680

[phy270993-bib-0055] Wills, T. A. , Soneji, S. S. , Choi, K. , Jaspers, I. , & Tam, E. K. (2021). E‐cigarette use and respiratory disorders: an integrative review of converging evidence from epidemiological and laboratory studies. European Respiratory Journal, 57, 1901815. 33154031 10.1183/13993003.01815-2019 PMC7817920

[phy270993-bib-0056] Wong, M. W. , Ross, N. A. , Chien, L.‐C. , & Bhammar, D. M. (2021). Respiratory and perceptual responses to high‐intensity interval exercise in obese adults. Medicine and Science in Sports and Exercise, 53, 1719–1728. 33587550 10.1249/MSS.0000000000002638

[phy270993-bib-0057] Zhou, Y. , Wu, F. , Deng, Z. , Wang, Z. , Tian, H. , Huang, P. , Zheng, Y. , Yang, H. , Zhao, N. , Dai, C. , Yang, C. , Yu, S. , Tian, J. , Cui, J. , Liu, S. , Wang, D. , Wang, X. , Lu, J. , Zhong, N. , & Ran, P. (2025). Lung function decline and incidence of chronic obstructive pulmonary disease in participants with spirometry‐defined small airway dysfunction: a 15‐year prospective cohort study in China. Respiratory Research, 26, 169. 40296032 10.1186/s12931-025-03244-3 PMC12039187

